# Development and Experiments of an Electrothermal Driven Deep-Sea Buoyancy Control Module

**DOI:** 10.3390/mi11111017

**Published:** 2020-11-19

**Authors:** Jiaoyi Hou, Weifeng Zou, Zihao Li, Yongjun Gong, Vitalii Burnashev, Dayong Ning

**Affiliations:** 1 National Center for International Research of Subsea Engineering Technology and Equipment, Dalian Maritime University, Dalian 116026, China; pohou@dlmu.edu.cn (J.H.); zwf.maple@foxmail.com (W.Z.); lzhmvp@foxmail.com (Z.L.); gyj@dlmu.edu.cn (Y.G.); 2 State Key Laboratory of Fluid Power and Mechatronic Systems, Zhejiang University, Hangzhou 310058, China; 3 Institute of Aerospace Technologies, National Technical University of Ukraine “Igor Sikorsky Kyiv Polytechnic Institute”, 03056 Kiev, Ukraine; vburnashev@mail.ru

**Keywords:** melting, condensation, phase transition, heat transfer, underwater robot

## Abstract

Due to the extremely high pressures in the deep sea, heavy ballast tanks and pressure compensating hydraulic tanks are typically required to support the operation of classic buoyancy controls. Buoyancy control systems driven by phase-change materials (PCM) have unique advantages over conventional hydraulically actuated buoyancy control systems, including high adaptability for deep-sea exploration and simple, lightweight, and compact structures. Inspired by this, a buoyancy control module (BCM) was designed with flexible material as the shell. Instead of a conventional mechanical system, the device uses an electric heating drive to control buoyancy by heating and cooling the PCM. Based on the principle of pressure compensation, this device can adjust the buoyancy of a small underwater vehicle in a deep-sea high-pressure environment. The BCM successfully adjusts the buoyancy to lift itself up and down in the South China Sea at a depth of 3223 m. The performance of the phase-change BCM to control buoyancy under high pressure is validated by systematic experiments and theoretical analysis. Our work proposes a flexible scheme for the design of a deep-sea phase-change-driven BCM and highlights its potential application in deep-sea micro-mechanical systems, especially soft robots.

## 1. Introduction

There has been increasing interest in ocean detection and submarine resources, many researchers are paying attention to developing underwater detection equipment, which can conduct various missions instead of humans in a hazardous environment. There are various types and forms of underwater detection equipment, but no matter what their forms and functions are, the device to regulate buoyancy is an essential subsystem of underwater detection equipment. There are two ways to control the depth of a submersible in water: power propulsion and adjusting buoyancy [1,2]. The first method is to drive the submersible with propeller thrust to control its vertical motion. Its suspension at a certain depth is achieved by controlling the combined force of gravity and buoyancy. Thus, the propeller must be in continuous operation during the submersible’s suspension. The underwater terrain is complex and supports a wide variety of aquatic plants and animals. Therefore, it is common for propellers to be entangled by underwater organisms, leading to failure. Additionally, the propeller keeps running during underwater depth adjustment, which leads to high energy consumption, great noise, and vibration. This method is generally not used alone and is often combined with the buoyancy control system to control the underwater vehicle.

Different from thrust-driven submersibles, the method of adjusting the net buoyancy of the equipment to achieve the depth adjustment of the submersible is more suitable for deep-sea submersibles with large water depth and long working hours. Net buoyancy means the combined force of buoyancy and gravity. There are two ways to adjust the net buoyancy, change its mass or change its volume of displacement. For example, the buoyancy regulation device of the throw-load type is a net buoyancy regulation way to reduce the equipment quality by discarding part of itself, whereas the use of a high-pressure sea pump to pump ballast water is another way to change its quality. It is widely used in the emergency floating devices of underwater vehicles. Another major category of buoyancy regulation is pumping and discharging ballast water or oil [3,4,5,6,7,8]. At present, the key device of this regulation mode is the deep-sea high-pressure pump, whose ultra-high pressure and lightweight manufacturing technology are still under development.

The throw-away buoyancy control can only be used once during a voyage and is generally only used when the submersible is recovered to the surface [9]. The two buoyancy regulation methods of seawater pump and oil bladder are necessary to carry a heavy driving motor and high-pressure valve control system. The equipment’s heavy weight and high noise limit its use in small submersibles. Moreover, the quality requirements of pumps and valves used in these devices for deep-sea buoyancy regulation are very precise and therefore difficult to manufacture. These factors limit its use in small deep-sea submersibles.

In recent years, scientists are exploring some novel phase-change buoyancy regulation devices, such as temperature difference variable buoyancy regulation [10], memory metal variable buoyancy regulation [11], and solid–liquid variable buoyancy regulation [12,13,14,15]. Phase-change buoyancy adjustment device has obvious advantages in the small underwater vehicle; therefore, we have noticed a new buoyancy regulation method based on paraffin phase transition. In the 1970s, Clarke proposed another hypothesis that insists that the spermaceti organ is used to control buoyancy by melting and congealing spermaceti oil [16]. A Japanese researcher named Koji Shibuya has developed a buoyance-regulating robot based on paraffin phase transitions [17].

The above buoyancy adjustment methods are various, but it is difficult to find a suitable method for the deep-sea work of small submersibles or micro-machinery. In recent years, the research of bionic underwater robots has developed rapidly, and many bionic fish robots have been developed [18,19]. Attaching flexible swim bladders to these flexible fish is an attractive research project.

Nature’s creatures, such as seals [20], larval anurans [21], and *Calanoides acutus* [22], etc., are often illuminating to scientific research, adjusting their buoyancy to move up and down in the water by changing their metrics of body density. Adjusting the buoyancy by changing the density of wax in the body is one of the most appropriate ways to adjust buoyancy in the deep sea, and the sperm whale has taken this adjustment to its extreme [16]. Inspired by this, we designed a phase-change buoyancy control module (BCM) with flexible material as the shell, highlighting their potential applications in deep-sea micro-mechanical systems, especially soft robots. Instead of the conventional mechanical drive, the BCM uses an electric heating drive to control buoyancy by heating and cooling the phase-change materials (PCM). Based on the principle of pressure compensation, the BCM achieves the goal of controlling the buoyancy of a small phase-change BCM in the deep sea. As the basis of this study, the melting process of paraffin was monitored with a thermocouple and the melting speed and state of paraffin driven by electric heating wire were observed. The BCM is suspended in the flume, and its buoyancy adjustment ability is verified by a force sensor, which detects the buoyancy change in its working process. The theoretical analysis and experiment show that there is good linearity between the buoyancy change and the heating time of the device, which indicates that it is easy to realize the precise control of the buoyancy with the device. The device directly converts electric energy into heat energy through the heating device and then transfers it to the phase-change material into internal energy. Our work proposes a flexible scheme for the design of deep-sea phase-change BCM and highlights their great potential for application in small deep-sea robots, especially for the buoyancy regulation of deep-sea flexible electronic fish.

## 2. Materials and Design

### 2.1. Thermal Expansion Capacity of Paraffin

**Figure 1:**
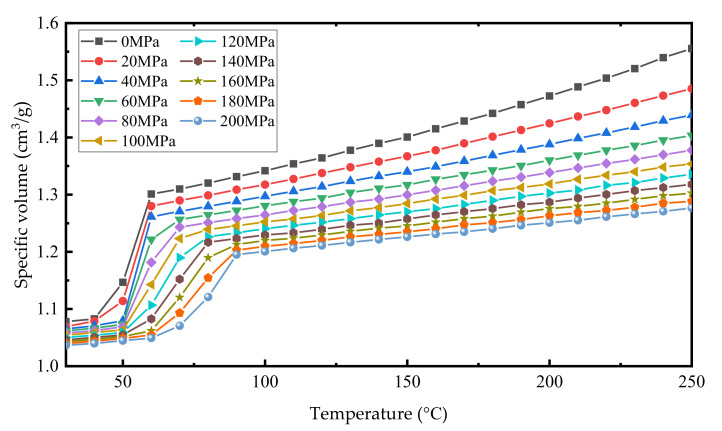
Specific volume for n-tetracosane (C_24_H_50_) as a function of temperature for pressures between 0 and 200 MPa. Data from Zoller and Walsh (1995).

The change of buoyancy in the phase-change buoyancy regulation system is caused by the change of drainage volume due to the change of the volume of the phase-change material. In particular, the volume change of PCMs with the increase of temperature is especially obvious during the solid–liquid state transition. Zoller and Srivastava conducted some related experiments on the properties of paraffin [23,24,25]. The results show that n-Tetracosane has a large volume change, typically 10–15%, coupled to the phase-change from solid to liquid, and the expansion is retained to a large extent even at pressures up to 200 MPa, Figure 1.

Studies by Koji Shibuya and Yukihiro et al. suggest that the volume change of the phase transition process of paraffin is 17% [14]. The density of PCM varies with temperature; even when the PCM is not undergoing a phase transition, which means that the BCM expands by far more than 17% of its PCM volume. According to Zoller and Srivastava, the volume of paraffin can vary as much as 43.5% when the temperature goes from 30 °C to 250 °C at a pressure of 0 MPa. In the design of the buoyancy adjustment device, we control the temperature of paraffin wax at 170 °C and the volume change does not exceed 32% to ensure the safety and stable performance of the device.

### 2.2. Module Design and Fabrication

**Figure 2:**
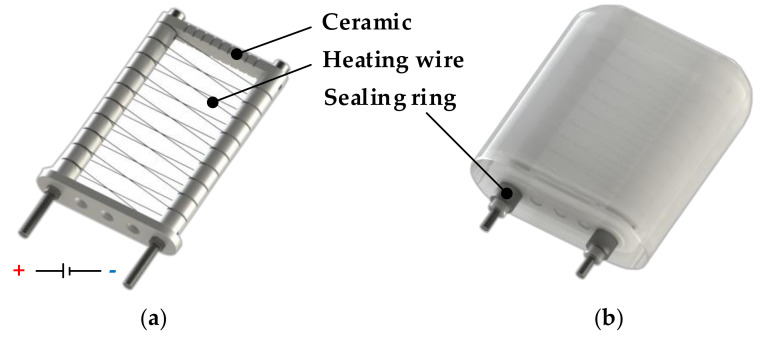
Design of heating device for paraffin: (**a**) heating wire, supporting structure, and insulation structure of heating wire; (**b**) the layout of the heating device in the buoyancy adjustment module and the leakage proof structure of the module.

The phase-change BCM is divided into two design units, the heating structure and the shell. A lightweight design and miniaturization are the design goals to pursue in order to achieve excellent performance. After several prototype productions and tests, we finally put forward a one-time molding design scheme with a silicone matrix as the shell to wrap the paraffin wax. Figure 2 shows the detailed structure of the heating unit and the overall layout scheme. The supporting structure of the heating wire is a combination of iron bolts and customized ceramic pieces of special structure. The detailed design of the heating unit is as follows:

1. The attachment structure of the heating wire is composed of a ceramic tube and bolt. A heating wire limit slot is machined on the ceramic tube for fixing the position of the heating wire.
2. These iron bolts not only act as a rack but also serve as electrodes for the heating wire. Inside the module, the two ends of the Ni-Cr alloy wire are welded to two bolts, respectively. The bolts exposed outside the module are also connected to the input electrode by welding.
3. The Ni-Cr alloy wire is wound on the ceramic insulating structure with a spiral structure and distributed evenly in the paraffin to obtain better heat transfer efficiency.
4. There is no adhesion between the exposed end of the bolt and the silicone base housing, and a clamping ring is placed on the housing in this position to prevent the paraffin from leaking when it is heated to a liquid during operation.

**Figure 3:**
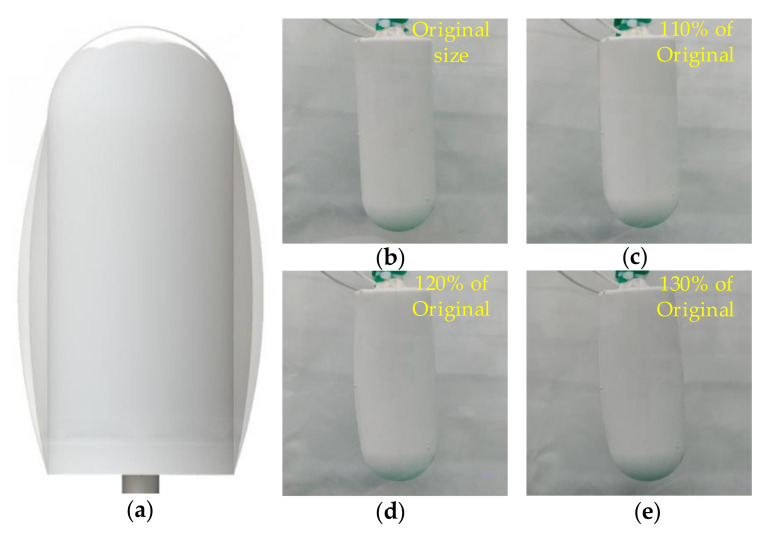
The form of buoyancy control module (BCM) in operation: (**a**) shape change; (**b**) original state; (**c**) 110% of original size; (**d**) 120% of original size; (**e**) 130% of original size.

The shape of the buoyancy adjustment module is designed as a flat semi-spindle. Compared with the spherical shape design, this design makes the shell of the BCM deform less when the paraffin wax is heated. One end of the screw in the heating unit protruded from the flat bottom of the module. The screw end is the power supply interface of the heating wire and can be used as the mounting bolt of the buoyancy adjustment module. Figure 3 shows the appearance change prediction of the buoyancy adjustment module during operation and appearance change in the actual test.

**Figure 4:**
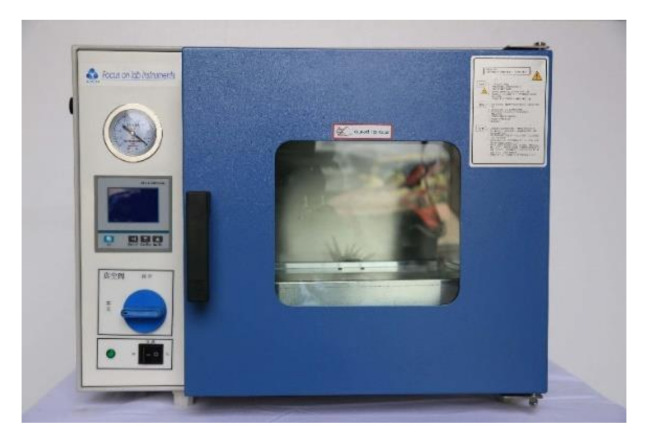
Vacuum chamber and high-temperature curing equipment.

Both the paraffin outline and the silicone shell are made by pouring. For this purpose, two sets of casting molds were designed and manufactured. The paraffin wax is melted by heating before casting, and the casting is in a high-temperature state. Therefore, the mold must be able to withstand high temperatures and not be prone to thermal deformation. The most important problem in the fabrication of phase-change buoyancy modulators is to inhibit the formation of the air gap in the paraffin and shell. Before pouring the paraffin, the assembled heating device and mold shall be preheated in a high-temperature drying oven. As shown in Figure 4, the vacuum drying box can be used for preheating metal molds and the vacuum-pumping of paraffin wax at high temperatures. This prevents the paraffin wax from cooling rapidly as it is poured into the mold at a low temperature. The rapid cooling of paraffin produces a large number of voids in the paraffin solids that it forms. When the BCM works under high pressure in the deep sea, the air in the void space is compressed, resulting in great buoyancy loss. When the paraffin does not melt in the deep-sea environment, the silicone shell will even crack due to the huge pressure difference.

**Figure 5:**
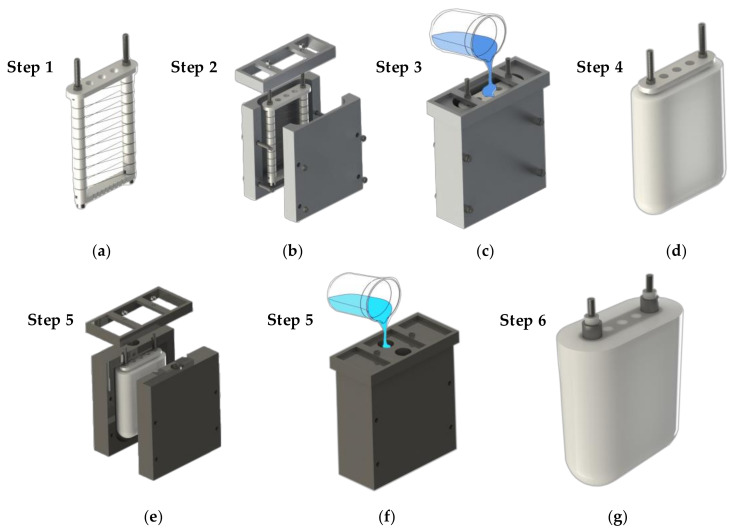
Fabrication process of paraffin phase-change BCM: (**a**) assembly of heating unit; (**b**) assembly of paraffin mold; (**c**) paraffin pouring; (**d**) paraffin mold release; (**e**) assembly of shell mold; (**f**) the silica gel components A and B are mixed and poured into the mold; (**g**) the finished module is removed after the silicone is cured.

The manufacturing process of the module is divided into heating unit manufacturing and the pouring and forming process of the paraffin wax and silicone shell. Figure 5 shows the detailed manufacturing process of the phase-change buoyancy adjustment module. The phase-change material used in the buoyancy adjustment device is industrial paraffin wax with a melting point close to 58 °C. This type of paraffin is a mixture of alkanes and is an inexpensive, non-toxic, and harmless material. The shell is made of AB mixed silicone with a Shore hardness of A15. The silica gel has moderate hardness, good flexibility, and ductility. At the same time, it has a long curing time at room temperature, which provides sufficient time for removing the air in silica gel during production. The final weight of the phase-change buoyancy regulator we produced is about 720 g, and the weight in water is about 80 g. The weight of the paraffin phase-change material filled in the BCM is approximately 170 g.

## 3. Experimental Procedures

### 3.1. Melting Experiment of Paraffin

**Figure 6:**
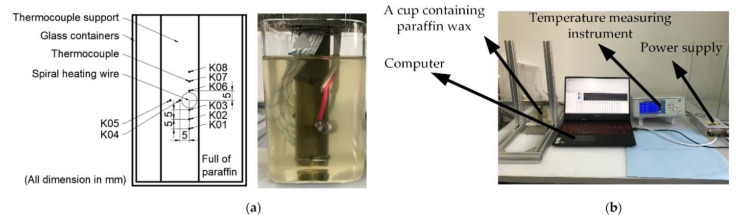
Design of heating device for paraffin: (**a**) heating wire and supporting structure and insulation structure of heating wire; (**b**) the layout of the heating device in the buoyancy adjustment module and the leakage proof structure of the module.

Reliable control of the phase-change process of paraffin is the key to control the buoyancy of the phase-change buoyancy regulator. Therefore, the experiment of heating wires to melt paraffin was carried out first. F.L. Tan et al. used water bath heating to heat paraffin and observed the melting of paraffin [26,27,28]. Vikram et al. performed numerical simulations of experiments that optimized F.L. Tan [29]. Their study showed that heat upwelling during paraffin melting had a significant effect on paraffin melting. To observe the melting process of paraffin during the active heating of the electric heating wire, we followed the experimental setup of F.L. Tan and used thermocouples to monitor the temperature of paraffin at different positions, as shown in Figure 6.

The multi-channel temperature acquisition system of the melting test experiment is shown in Figure 6a. The experimental equipment includes a 0–48 V adjustable power supply, a square glass, eight Type K thermocouples, a computer, and a multiplex temperature acquisition device. The computer collects real-time temperature data of eight thermocouples through the temperature acquisition device. Figure 6b shows the placement of eight thermocouples, which are arranged around the spiral heating wire. Among them, the diameter of the spiral heating wire contour is 8 mm, and the distance from K03, K04, and K06 to the center of the heating wire contour is 5 mm. The distance between the two adjacent K-type thermocouples is also 5 mm. The thermocouple and spiral heating wire are arranged on the bamboo holder to ensure that they do not shift. The power of the heating wire is controlled by the adjustable power supply, and the data collected by the thermocouple are transmitted to the multi-channel temperature acquisition instrument and finally to the computer for recording, sorting, and analysis. The melting process of paraffin can be reflected by analyzing the temperature variation of thermocouple in different positions.

### 3.2. Buoyancy Testing

It is difficult to directly measure the buoyancy change of the BCM in water. The weight of the prototype we made is about 80 g in water, and the buoyancy of the prototype can be calculated by monitoring the weight change of the prototype in water during paraffin heating. We suspended the prototype in water through a force sensor that measured the combined force of gravity and buoyancy that the prototype was subjected to, which we labeled as equal to the weight of the prototype in water and decreases as the buoyancy increases.

**Figure 7:**
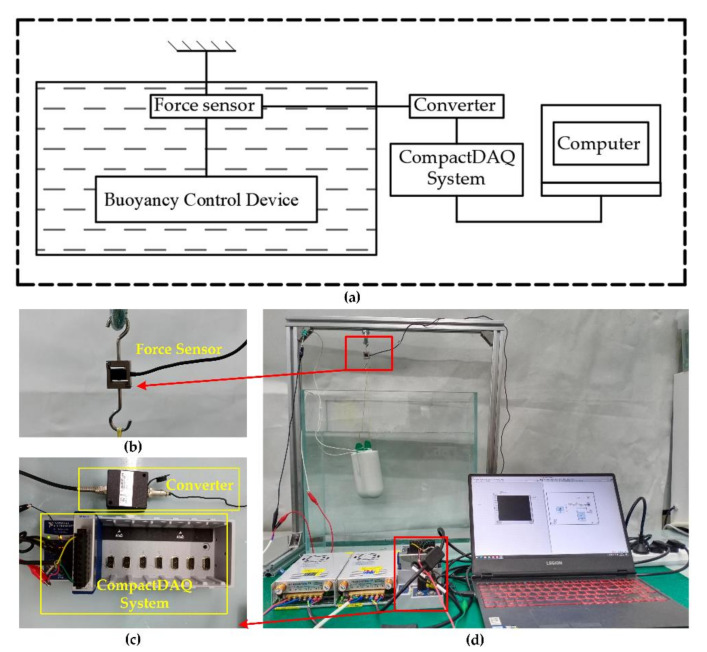
Buoyancy adjustment ability test bench: (**a**) schematic diagram of buoyancy testing system; (**b**) tension sensor (range 0–4.9 N); (**c**) CompactDAQ system; (**d**) buoyancy adjustment performance test site.

Figure 7 shows the principle of the buoyancy test system and some experimental equipment. The tension data collected by the force sensor are analyzed and recorded by NI’s CompactDAQ through the signal converter, and finally stored in the computer. Table 1 shows the test experimental parameters not mentioned above.

### 3.3. Rise and Levitation

#### 3.3.1. Rise and Levitate in the Tank

**Figure 8:**
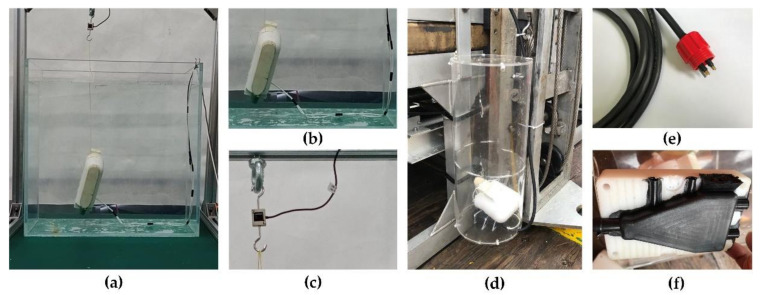
Experimental design and installation details for the rise and levitation tests: (**a**) experimental layout in the flume (0 MPa); (**b**) power supply wire; (**c**) force sensor (real-time monitoring of net buoyancy when net buoyancy is less than 0); (**d**) the installation position of the buoyancy adjustment device on the Seahorse; (**e**) deep-sea cable watertight joint; (**f**) curing treatment of deep-sea cable watertight joints.

The ultimate goal of designing a phase-change BCM is to provide a new kind of buoyancy control subsystem for a small underwater vehicle. Therefore, we conducted a series of experiments to prove that it can be competent for the work of buoyancy regulation. We adjust the net buoyancy by installing the buoyancy block on the prototype. First, we carried out tests in the tank under atmospheric pressure and set the net buoyancy to minus 5 g. The prototype is suspended in the tank by tension sensors and thin wires, and its buoyancy changes can be accurately measured in real time. Figure 8a–c shows the installation details of the test system. The adjustable power supply is used to supply power to the prototype and the net buoyancy change and motion state of the prototype are observed in real time.

#### 3.3.2. Deep-Sea Tests in the South China Sea

In mid-August 2020, we took the prototype to the South China Sea for a deep-sea verification test. The prototype was carried to the bottom of the South China Sea at a depth of 3223 m by a 6000-m deep-sea ROV Haima 2. The BCM is placed in a protective cover made of acrylic material to prevent the prototype from being damaged by the sea waves as it dives. In deep-sea tests, the prototype was powered by Haima 2. After the prototype arrives at the bottom of the sea and settles down smoothly, the power is switched on. The deep sea is harsh, with a temperature of only about 3 degrees Celsius and strong corrosion and electrical conductivity. The deep-sea cable uses the special joint and vulcanizes the welding point of the wire. The circuit of the prototype must be well insulated to avoid short circuits due to leakage. Figure 8d–f shows the installation position and cable of the buoyancy adjustment module on Haima 2.

## 4. Results

### 4.1. The Process of Electroheating Paraffin Phase-Change

**Figure 9:**
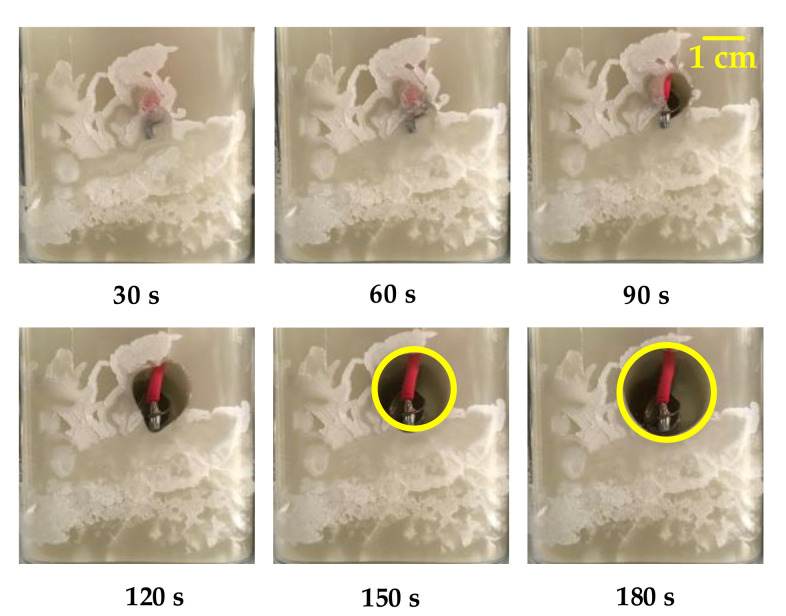
Melting process (0–180 s at 50 W power).

**Figure 10:**
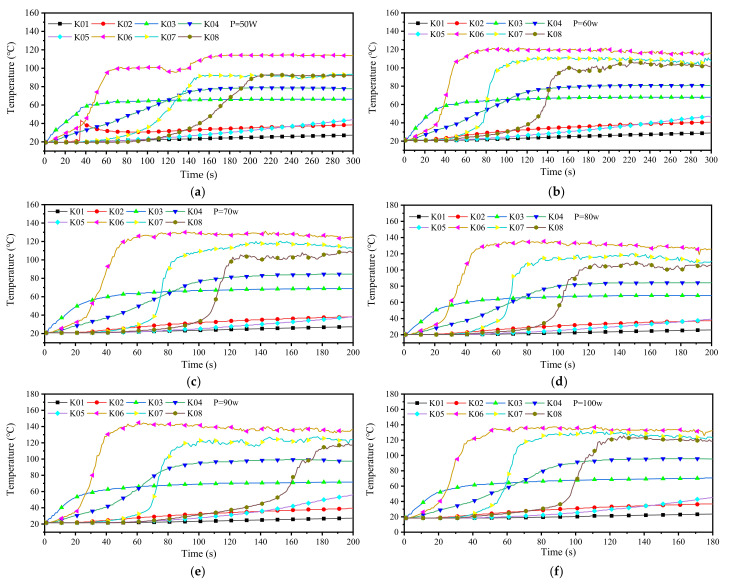
Monitoring the melting process of paraffin: (**a**–**f**) the temperature of each position when the heating power is 50, 60, 70, 80, 90, and 100 w.

**Figure 11:**
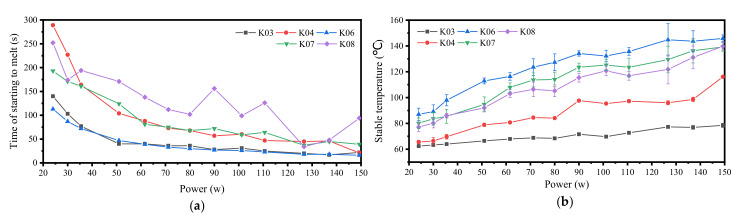
Summary of phenomena in the melting process of paraffin: (**a**) at different power, the heating time of each position starting to melt; (**b**) the stable temperature of each position.

The paraffin in our study was heated by electric heating wires to melt. Different from the isothermal wall surface heated by a water bath, the heat power of the heating wire is constant. The liquid region formed by our melting experiment is an eccentric circle with the center of the spiral heating wire as the center. When the heating power is 50 W, the formation process of the liquid region is shown in Figure 9. After heating 180 s, the cross-section diameter of the liquid region formed is 20 mm. Morphologically, the effective heating area of the heating wire with a diameter of 8 mm under this power is an eccentric circle with a diameter of 20 mm. The following conclusions can be drawn from the temperature data recorded by the multi-channel temperature recorder:

1. At the beginning of paraffin melting, the heat transfer form between the heating wire and paraffin is mainly heat conduction. After the liquid region is formed, the heated liquid paraffin expands in volume and decreases in density. The liquid paraffin expands along the holes or gaps around the molten pool, which may lead to sudden local temperature rise (as shown in Figure 9, under the condition of *P* = 50 W, when the heating time is 30 s, the temperature curve at K02 changes suddenly). After the molten pool is formed, the heated liquid paraffin generates a thermal upwelling driven by buoyancy due to its reduced density. This kind of movement inside the molten pool makes the heat in the molten pool continuously transferred to the upper part, making the upper part of the molten pool hotter than the lower part. Therefore, the solid paraffin on top of the molten pool continues to melt, whereas the paraffin on the bottom does not melt easily.
2. In the process of melting after being heated by the electric heating wire, the paraffin will gradually form a stable temperature field, and the temperature of each position will fluctuate in a small range. Regions that have reached a stable temperature during the continuous melting process will not experience a significant increase in temperature.
3. The stable temperature field will be disturbed by the irregular thermal upwelling, and the irregular thermal upwelling in paraffin will break the original equilibrium and form a new stable temperature field, Figure 10.
4. With the increase of heating power, the steady-state temperature of paraffin increases, and the time from heating to melting is shortened, Figure 11.

Some constructive conclusions were drawn by analyzing the melting phenomena and temperature changes at each point in the process of paraffin melting. First, the higher the temperature, the lower the density in the liquid paraffin formed by the melting of solid paraffin. Because of the density difference in the liquid paraffin, the molten paraffin will form a significant upward thermal flow. This heat flow will promote the heat exchange in paraffin and increase the heat transfer rate. On the contrary, the heat flow hindered by obstacles will slow down the speed of heat transfer. Another concern is that because of the poor thermal conductivity of paraffin, the farther the example between the solid paraffin and the heating wire is, the more difficult it is to melt. Only the paraffin within 5 mm of the heating wire has a faster melting speed, whereas the melting of the paraffin at a farther distance mainly depends on the heat flow.

### 4.2. Buoyancy Regulation Performance

The test of buoyancy regulation performance in the water tank verified that the phase-change buoyancy regulation prototype produced by us had controllable buoyancy regulation capability. The conclusion that the volume change of paraffin phase-change is 17% is presented in the paraffin phase-change buoyancy adjusting robot developed by Ryukoku University in Japan. However, the change of buoyancy in a PCM engine is caused not only by the phase transition of PCM but also by the continuous decrease of the density of PCM as the temperature increases. When heated from 20 °C to 200 °C, the volume of paraffin wax expands to 136.9% of its original volume. That is to say, when the temperature of paraffin wax rises from 20 °C to 200 °C, the buoyancy regulation ability reaches 36.9%. This was confirmed in our buoyancy test.

**Figure 12:**
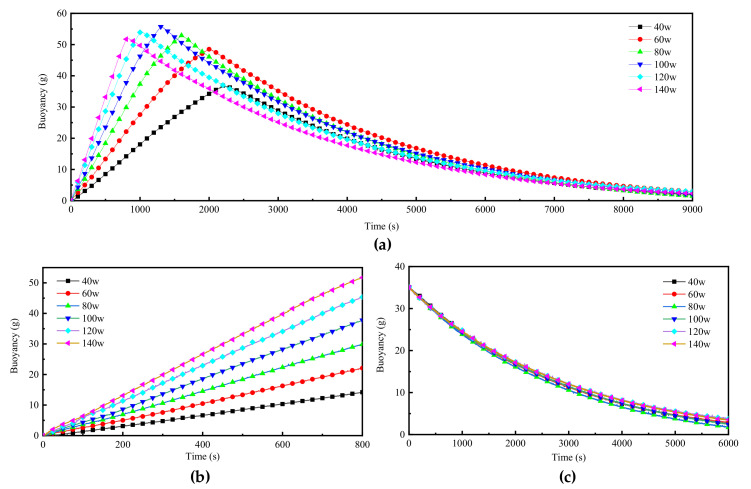
Experimental results of BCM in flume: (**a**) the buoyancy change monitoring of the BCM under different power; (**b**) buoyancy change monitoring of BCM running under different power within 800 s; (**c**) drop curve of buoyancy starting from 35 g under the natural cooling state.

In our test, when the driving power is 140 W, it only takes about 435 s for the increased buoyancy value to reach 17% of the PCM volume. At 800 s, the added buoyancy reached 30% of the volume, and the power had to be turned off to prevent safety problems caused by the excessively high temperature of paraffin wax. Figure 12a shows the whole-course buoyancy monitoring data of the six tests (to visualize the buoyancy, the units are converted to grams). In the case of a larger power, the growth rate of buoyancy does not decrease significantly after the maximum increment of buoyancy reaches 35%. This indicates that the adjustment ability of the buoyancy adjustment module tested does not reach the limit value. However, to ensure the safety of the device and avoid the explosion of PCM due to overheating, it is suggested that the adjustment range of PCM buoyancy should be limited to 37% of the material volume and 200 °C of the material temperature.

**Figure 13:**
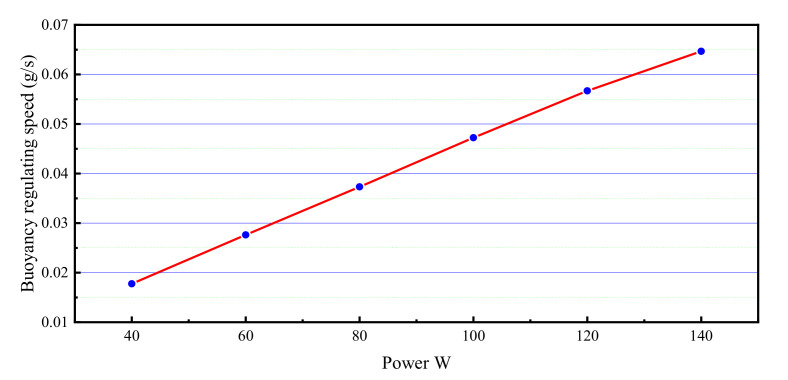
The buoyancy regulation speed of BCM under different driving power.

We extracted the data of buoyancy value during the 800 s when the buoyancy adjustment module was switched on to form a separate statistical report. It is easy to observe that no matter what the driving power, the buoyancy value and the operating time of the device can form a high linear correlation. The buoyancy regulation speed is obtained by dividing the buoyancy by the time of continuous heating of the buoyancy regulator, as shown in Figure 13. When the driving power is 140 W, the regulating speed reaches 0.065 g/s. It is also found that there is a high linear correlation between the buoyancy regulation speed and heating power. These results indicate that the phase-change buoyancy regulator is expected to have the same controllability as other modes of buoyancy regulation.

The cooling mode of our phase-change buoyancy regulator is natural cooling. The reason for this design is to simplify the structure of the device as much as possible and to simplify the working principle of the system to adapt to the harsh environment of low temperature and high pressure in the deep sea. Figure 12c is obtained by sorting out the buoyancy data recorded in the next 6000 s when the buoyancy is reduced to 35 g during the cooling process. According to the data in the figure, the buoyancy decline rate under different driving powers in 2000 s was almost the same. As time goes on, the temperature of the material decreases, and the buoyancy decreases slowly.

### 4.3. The Independent Rise and Fall of BCM

In the experiment of buoyancy monitoring, the buoyancy regulation ability of the phase-change BCM has been proved. In this part, we realized the control of its heave motion in the pressure of 0 MPa and the deep sea with a pressure of 32 MPa.

**Figure 14:**
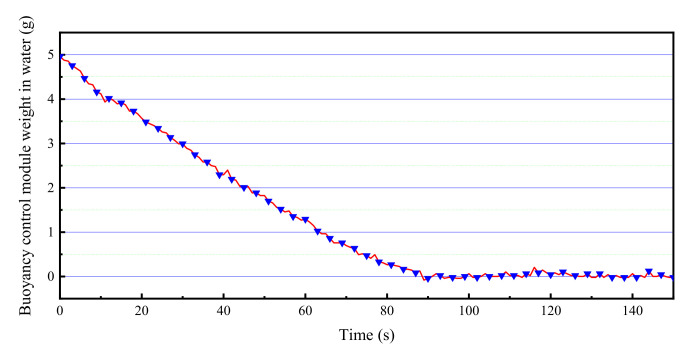
The weight of BCM in water.

**Figure 15:**
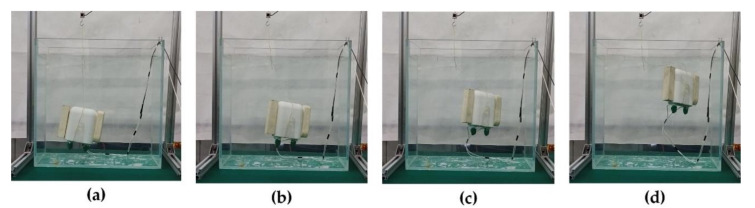
The phase-change BCM is driven by the buoyancy to control the rise and suspension in the tank: (**a**) initial state (tension shown by force sensor is 4.5 g); (**b**) buoyancy is greater than zero, and it starts to rise; (**c**) stop heating and hover in the water; (**d**) continue heating and rise to the surface.

In the water tank, the prototype was controlled by the driving power of the controller to perform fast floating and hovering respectively. We gave the prototype of the BCM a constant power of 140 W. Within 90 s of the start of heating, the weight of the BCM in water is reduced from 5 to 0 g. Figure 14 is the real-time monitoring data of buoyancy changes in the experiment. When the heating time was 90 s, it was observed that the prototype began to accelerate and rise in the water, and it surfaced when the heating time was 110 s. It rose 0.19 m in 20 s. We also realize the levitation of the buoyancy regulating the device in water by adjusting the heating power. Figure 15 shows the suspension control process. These experiments further confirm that the phase-change BCM we developed has excellent performance.

**Figure 16:**
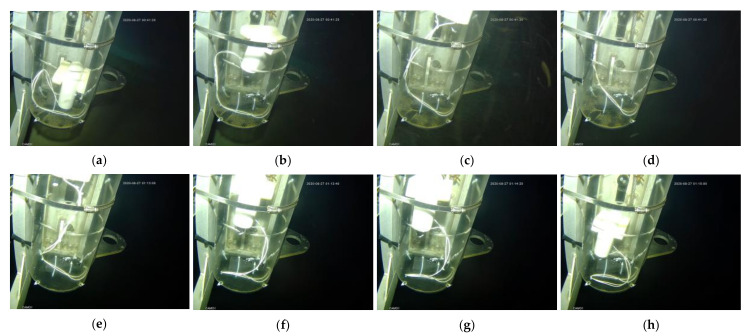
The phase-change BCM is capable of autonomous uplift and subsidence at a depth of 3223 m in the South China sea: (**a**–**d**) rising process; (**e**–**h**) descent process.

In the South China Sea, it takes an hour and a half for an ROV with our prototype to reach the depth of 3000 m and the same time for the recovery to come ashore. Considering the uncertainties of deep-sea environmental experiments, the prototype was given a small power drive of 23 W in the verification test of the rise and sink control performance in the deep-sea environment. Although it’s speed of buoyancy regulation became slower at low power operation, it completed its autonomous ascent in a deep ocean environment of 3223 m. It also managed to sink to the bottom of the shield after the power cut. Figure 16 shows the ascent and descent of a prototype in a protective casing in deep water.

## 5. Discussion

PCM has been widely studied and applied in many fields, such as energy storage materials [30] and driving. Especially for actuators in micromechanical applications [31,32,33] and even as a new material for aerospace applications [34]. PCM is widely used but rarely used in the field of deep-sea buoyancy regulation. Inspiration from micro-actuators in the field of micromechanics led us to the idea of developing micro-buoyancy regulation devices in the deep sea.

In this paper, a phase-change BCM based on paraffin phase-change material is proposed. At a high pressure of 100 MPa, the thermal volume expansion of paraffin can reach 20%. This means that the paraffin phase-change BCM is capable of excellent buoyancy regulation at a depth of 10,000 m, and this performance can be achieved only by giving it enough heat. This could be an ideal way for researchers working with small robots in the deep ocean to adjust their buoyancy. At the same time, we propose a phase-change BCM structure based on the principle of pressure compensation, which replaces the bulky mechanical protective shell with the soft and light silicone shell. Silicone has excellent ductility and heat insulation, but during the preparation process of the device, the air in the silica gel needs to be removed from the vacuum chamber to reduce its buoyancy loss under the high-pressure deep-sea environment. Similarly, the air in paraffin needs to be removed in this way, except that the air extraction treatment of paraffin requires the whole process to be carried out in an environment above the melting point.

There are several other things worth noting and discussing during the production process, specifically as follows:

1. Special attention should be paid to the welding reliability between Ni-Cr heating wire and electrode in the fabrication of the heating unit. Weak welding will make the buoyancy adjustment module produced short-circuit fault in the process of use.
2. The metal mold must be preheated to above the melting point of paraffin when pouring the paraffin. Otherwise, the liquid paraffin will cool down quickly when pouring into the mold. The paraffin solids formed in this way will be mixed with many bubbles, which will seriously affect the quality of the finished product, resulting in waste products.
3. Vacuum operation at high temperatures after pouring paraffin can reduce the bubbles in solid paraffin. The slow cooling of paraffin and the avoidance of supercooling caused by the temperature drop can also significantly improve the forming quality of paraffin solids.
4. The curing process of silica gel should not be accelerated in a high-temperature environment. At high temperatures, the morphology of paraffin will change uncontrollably, and the product quality is better with the method of curing at room temperature.

The total weight of the prototype we produced is 720 g and the adjustment capacity reaches 55 g. When a very small autonomous underwater vehicle (AUV) adjusts its buoyancy in this way, the total weight of the buoyancy adjustment unit can be reduced to 20 g if it only needs 3 g of buoyancy adjustment capability. The following work will carry out the theoretical research of the phase-change buoyancy regulation device: the exploration of its control mode, including electric heating active phase-change material melting process numerical simulation, and the study of its buoyancy loss under high-pressure conditions in the deep sea.

## 6. Conclusions

Through a series of experimental studies, the control mode of the phase transition generated by electrothermal driving phase-change materials is observed and analyzed. On this basis, a novel phase-change buoyancy regulator is designed and manufactured in this paper. The functional test and performance test of the system verify its ability of buoyancy adjustment. The test of the buoyancy regulation of the prototype shows that there is a good linear correlation between the change of buoyancy and the electrification time. This indicates that it has the same controllability as the traditional hydraulic buoyancy regulation system. Compared with the traditional buoyancy regulation system, it has an excellent lightweight performance. A prototype phase-change buoyancy control module has been successfully raised and lowered 3223 m to the seabed of the South China Sea. In this paper, we propose a new type of buoyancy control method for deep-sea robots, which has some enlightening significance for the buoyancy control method of deep-sea micro- and small equipment.

## Figures and Tables

**Table 1:** Parameters of the test system.

Parameter	Symbol	Values
Physical dimensions of BCM	*/*	(*mm*)
Weight of prototype in air	*G_a_*	720 g
Weight of the PCM filled in	*m*	170 g
Weight of BCM in water	*G_w_*	80 g
Range of tension sensor	*F*	0–4.9 N
Environmental stress	*P*_1_	0.1 MPa
Environment temperature	*T*_0_	20 °C
Voltage range of the power supply for heating	*U*	0–48 V
Data acquisition frequency	*/*	1 Hz
